# Biological responses to terahertz radiation with different power density in primary hippocampal neurons

**DOI:** 10.1371/journal.pone.0267064

**Published:** 2023-01-20

**Authors:** Li Zhao, Ruhan Yi, Sun Liu, Yunliang Chi, Shengzhi Tan, Ji Dong, Hui Wang, Jing Zhang, Haoyu Wang, Xinping Xu, Binwei Yao, Bo Wang, Ruiyun Peng

**Affiliations:** 1 Beijing Institute of Radiation Medicine, Beijing, PR China; 2 Central Laboratory, Hainan General Hospital, Hainan Affiliated Hospital of Hainan Medical University, Haikou, Hainan, PR China; Joint institute for High Temperatures of the RAS, RUSSIAN FEDERATION

## Abstract

Terahertz (THz) radiation is a valuable imaging and sensing tool which is widely used in industry and medicine. However, it biological effects including genotoxicity and cytotoxicity are lacking of research, particularly on the nervous system. In this study, we investigated how terahertz radiation with 10mW (0.12 THz) and 50 mW (0.157 THz) would affect the morphology, cell growth and function of rat hippocampal neurons in vitro.

## Introduction

Terahertz waves are non-ionizing radiation with frequencies ranging from 0.1 to 10 THz and have wavelengths between 3 mm and 30 μm. Terahertz waves have been emerged as valuable tools in various fields, such as biomedical imaging, industrial quality control, defense and security [1], due to its unique properties. Firstly, terahertz waves can penetrate through a wide variety of materials, such as skin, fabric, plastic and wood. Secondly, many particles including biomolecules such as proteins and DNA have characteristic absorption lines at frequencies within terahertz range. Thirdly, terahertz radiation is highly reflected by metals.

Recent years, more and more groups are interested in exploring the biological effects of terahertz radiation, most of which focused on fibroblasts and lymphocytes [2, 3]. However, the biological effects and mechanisms of terahertz radiation on neuronal cells and nervous system are still largely unexplored. Contradictory results are always been reported, due to different parameters, such as frequency and power intensity. Bourne *et al*. reported that 0.14 THz radiation with intensity between 24 and 62 mW/cm^2^ does not produce detectable adverse effects in neuronal cell line [4]. In primary neurons, it has been reported that low power terahertz radiation does not cause detectable effect, while high power radiation injured membrane potential [5]. Moreover, terahertz radiation also could increase level of depression to reduce neuronal activities [6]. However, other studies also demonstrated that indicated terahertz waves could improve the neuronal activities, such as enhancing the permeability of voltage-gated calcium channels [3], elevating intracellular Ca^2+^ concentration in neurons [7], increasing the content of Glu and decreasing the content of Ala in primary cortical neurons [8], and promoting the growth of ganglion processes [9].

In this study, we aimed to investigate terahertz caused biological effects in rat’s primary hippocampal neurons. We found that terahertz radiation did not produce significant effects on neuron growth and differentiation. However, terahertz with two different power intensities differentially affected cellular activity and apoptosis. Our results indicated that the effects of terahertz radiation on hippocampal neuron depended on parameters of terahertz radiation, such as frequency, power, exposure time as well as the measurement time.

## Materials and methods

### Primary rat hippocampal neurons

Primary hippocampal neuronal cells were isolated according to the published protocols [10]. Briefly, newborn Wistar rat (SPF Beijing biotechnology co., LTD) pups were euthanized within 12 h after birth and sterilized briefly with 75% ethanol. Cranium of the pup was opened using sterile scissors and the entire brain was removed. The brain was rinsed with cold tissue dissociation buffer (HEPES with 0.3% glucose, 0.75% sucrose, 320–330 mOsm, pH 7.2–7.4). Meninges surrounding the hippocampus was removed with forceps. The isolated hippocampus was gently minced in a tissue culture dish. The minced tissue was then transferred to HBSS buffer containing 0.25% Trypsin and incubated at 37°C for 15 to 20 min with gentle shaking every 5 min. Digested tissue was centrifuged for 5 min at 150 g. The supernatant was removed and cells were counted using a hemacytometer. The experiments and corresponding procedures were approved by the ethics committee from Beijing Institute of Radiation Medicine.

Primary rat hippocampal neurons were cultured in DMEM medium with 10% FBS, 10% horse serum and 1% L-Glutamine at a density of 5×10^5^/ml. Four hours later, the medium was changed to DMEM plus 10% horse serum, 1% N-2, 2% B-27, 1% L-Glutamine. Ara-C (5 μg/ml) was added to the medium after 24 hours’ culture, and 50% of the total medium was changed every 24 h from then on. Neurons isolated were randomly divided into different groups.

### Terahertz radiation

In vitro exposure was performed using Terahertz Wave Bio-Exposure system (TWBEs) from Beijing Institute of Radiation Medicine [11]. Terahertz waves from a terahertz source were emitted into the free space at a certain divergence angle. The terahertz lens was coaxial with the transmitting port of the terahertz source. After adjustment of the distance between the terahertz lens and the transmitting port of the terahertz source, the terahertz wave after passing through the terahertz lens was a parallel terahertz beam, and the neurons attached to the petri dish were radiated from bottom to top. The frequencies and power were 10mW (0.12 THz) and 50 mW (0.157 THz). The radiation periods were 10 min and 30 min respectively. Cells in sham-irradiated group were processed as that in terahertz exposed group, but without radiation.

### Cellular activity assay

The conversion of tetrazolium salt (WST-8) to water-soluble WST-8 formazan is dependent on the activity of cellular dehydrogenase. Therefore, Cell Counting Kit-8 (CCK8) can be used to determine the cellular activity via WST-8/1-Methoxy PMS system. In this study, the cellular activity was analyzed by cell counting kit-8 (CCK-8) (Dojindo Molecular Technologies, Shanghai, China) according to the manufacturer’s protocol. Briefly, rat primary hippocampal neuron was plated in a 96-well plate at a density of 1×10^3^/100 μl per well. CCK-8 was added to each well either immediately after radiation or 1 h after terahertz radiation. Cells were further incubated at 37°C for 4 h and absorbance at 450 nm was measured.

### Cellular apoptosis

Immediately after exposure to various terahertz radiation, the cellular apoptosis was analyzed by Annexin V staining (Beijing Biosea Biotechnology, Beijing, China), using un-exposed cells as control. Briefly, cells were washed and resuspended in 100 μl binding buffer. Each sample was incubated with 5 μl Annexin V-APC at the room temperature for 10 min followed by incubation with 5 μl propidium iodide for 5 min. Then, 400 μl PBS was added and the cellular apoptosis were analyzed by flow cytometer (FACSCalibur, Bio-Rad, USA). And, the percentage of apoptotic cells among total cells, including AnnexinV^+^PI^-^ cells and AnnexinV^+^PI^+^ cells were calculated.

### Transmission electron microscopy

Cultured hippocampal neurons were harvested by centrifugation immediately after terahertz radiation. Cells (1×10^6^/ml) were fixed in a solution containing 2.5% glutaraldehyde for 2 h followed by another 2 h fixation with 1% osmium tetroxide. Specimens were dehydrated by ethanol and acetone and embedded with Epon812. Ultrathin sections (70 nm) were made and stained with uranyl acetate and lead citrate. The cellular structure was observed by a transmission electron microscope (HITACHI H7650, Japan).

### Quantification of amino acid neurotransmitter by HPLC

Immediately after terahertz radiation, 500 μl cell medium was aspirated and centrifuged at 3000 rpm for 10min at 4°C. The supernatant was aliquoted and stored at 4°C. The concentrations of amino acid in the supernatant, including Ala, Gly, Phe, His, Ser, Val, Lue, Ile, Lys, Arg, Thr, Met, Pro, Glu, Tyr and Cyss were measured by high performance liquid chromatography (HPLC) (Hewlett Packard, USA). The amounts were calculated based on standard solutions.

### Detection of PSD-95 by immunofluorescence

Cells were fixed using 4% paraformaldehyde in PBS (pH 7.4) for 10 min at room temperature and washed 3 times with cold PBS, followed by permeabilization by 0.2% Triton X-100 for 10–15 min at room temperature. Cells were incubated with PBST-BSA buffer (TBS with 0.1% Tween-20 and 3% BSA) for 30min at 37°C, and then the anti-PSD-95 antibody (Rabbit monoclonal, 1:1000, Sigma, USA) was added to the coverslip. After incubation at 4°C overnight, samples were washed 3 times with PBST, 5 min each wash. After incubated with diluted secondary antibody (1:200 in PBST) at room temperature for 45 min and 3 times wash with PBST, the coverslip was sealed with DAPI mounting medium (Sigma, USA). Slides were examined under Radiance 2100TM confocal microscope (Bio-Rad, USA).

### Statistical analysis

All data were expressed as (mean±s.e.m). Statistical analysis was performed using SPSS18.0 software. The statistical analysis used two-tailed t-test for two groups, and one-way ANOVA followed by t-test for multiple groups. The difference with p<0.05 was considered as statistically significant.

## Results

### Terahertz radiation alter the activity of primary hippocampal neuron depending on power density and exposure period

**Fig 1:**
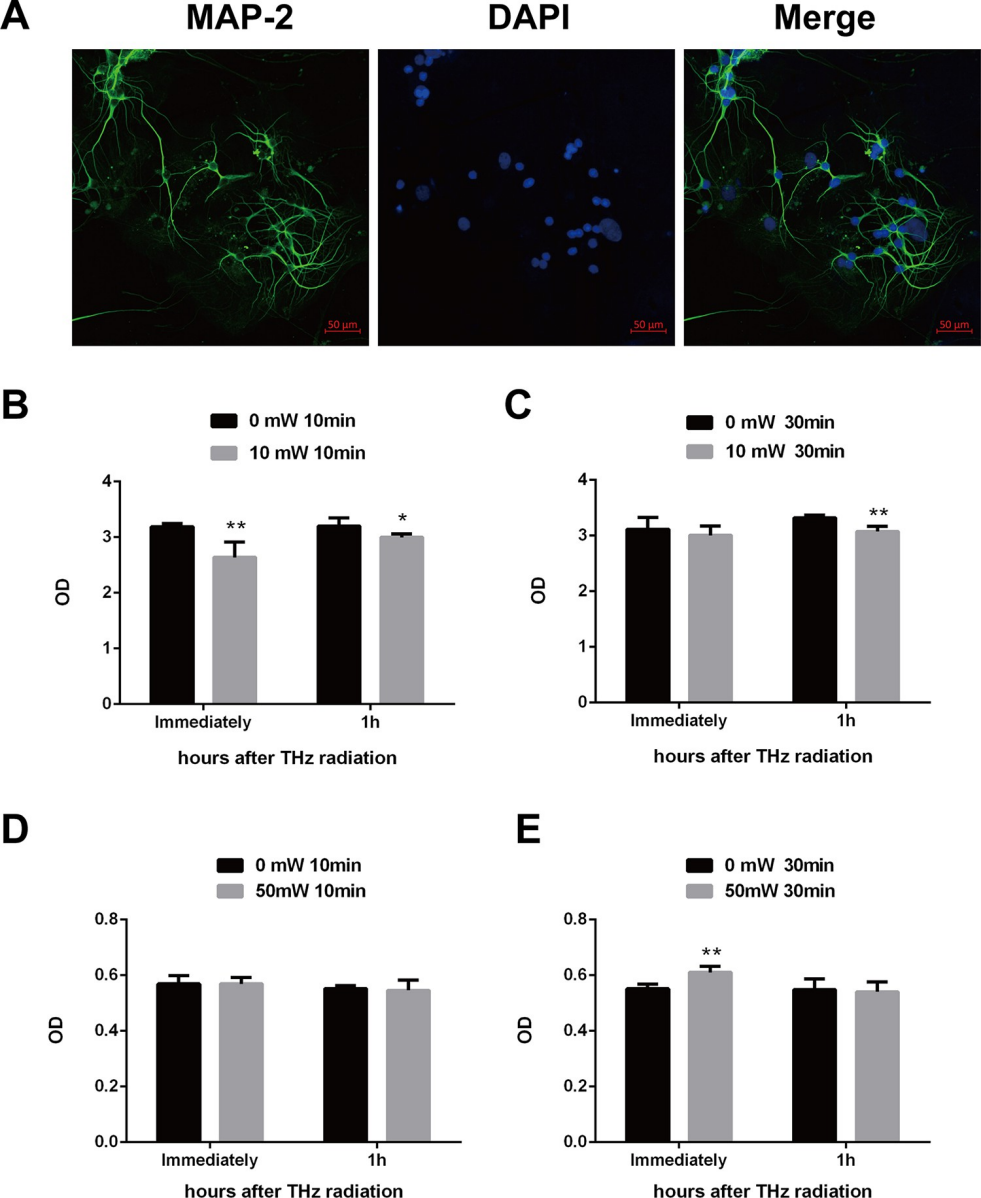
Effect of terahertz radiation on activity of the primary hippocampal neuron. Primary rat hippocampal neurons were isolated and cultured as described in materials and methods. **(A),** primary hippocampal cells showing neuronal staining with MAP2 (green). Nuclei were stained with DAPI (blue) (scale bar = 50 μm). Cellular activity was measured by CCK-8 assay immediately or 1 h after indicated terahertz radiation. The cellular activities were shown in B and C after exposure to 0.12 THz with power of 10 mW for 10 min and 30 min, respectively. Moreover, cellular activities were also presented in D and E after radiated by 0.157 THz with power of 50 mW for 10 min and 30 min respectively. Sham-radiated cells under the same conditions were used as the controls. Three independent experiments were conducted. Data were shown as mean±s.e.m. *p<0.05, **p<0.01 vs corresponding group.

Hippocampus is critical for memory and learning, and the potential effects of terahertz radiation on rat hippocampal neurons were investigated in this study. The hippocampal neurons were identified by observing morphological characteristics and detecting MAP2 protein. MAP2 protein is a neuron-specific, microtubule-associated protein which could promote the assembly and stability of the microtubule network, and has been emerged as a marker of neuronal cells [12]. And, typical neuronal morphology could be detected by MAP2 staining (Fig 1A). The cellular activities were analyzed by cell counting kit-8 (CCK-8) assay in primary hippocampal neurons after exposure to various terahertz radiation. Compared to sham-radiated cells, the cellular activity was significantly decreased immediately and 1 h after exposure to 0.12 THz with power of 10 mW for 10 min (P<0.01) (Fig 1B). Similarly, results were observed in primary cells after exposure to 0.12 THz with power of 10 mW for 30 min (P<0.01) (Fig 1C). Moreover, the cellular activity of primary cells increased obviously after exposure to 0.157 THz with power of 50 mW for 30 min (P<0.01), but not for 10 min (Fig 1D and 1E). Because exposure to 10mW (0.12 THz) and 50 mW (0.157 THz) for 30 min exerted opposite effects on cellular activity, we investigate the effects of terahertz on primary hippocampal neurons by 30 min’s radiation.

### Terahertz radiation induced the cellular apoptosis in primary hippocampal neuron

**Fig 2:**
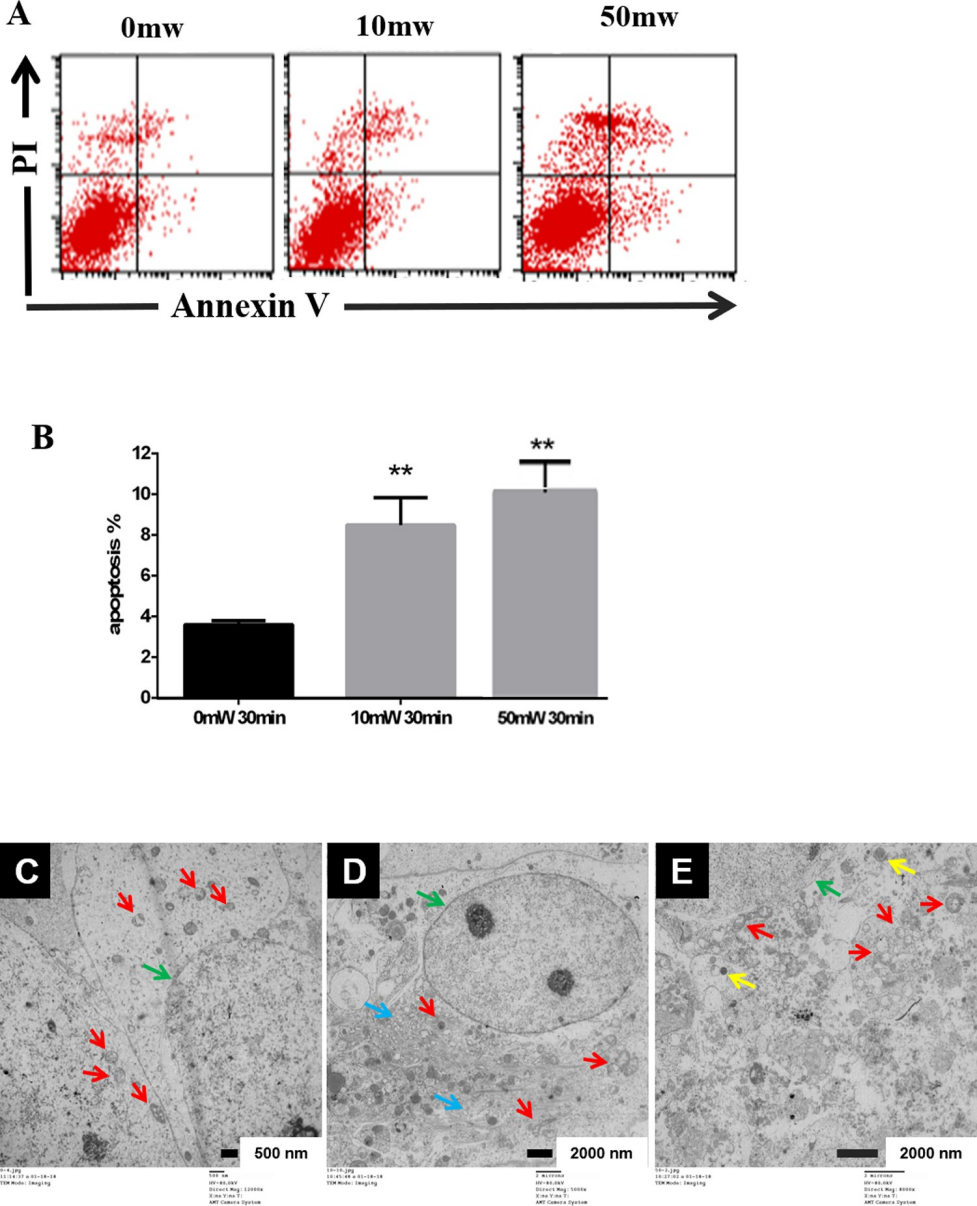
Effect of terahertz radiation on the apoptosis and ultrastruture of primary hippocampal neuron. Rat hippocampal neurons were radiated by 0.12 THz with power of 10 mW for 30 min or by 0.157 THz with power of 50 mW for 30 min. Apoptosis was analyzed by flow cytometry using Annexin V and propidium iodide staining immediately after terahertz radiation. The representative images were shown in **A**, and the statistical analysis were shown in B. Sham-irradiated cells were used as the controls. Three independent experiments were conducted. Data were shown as mean±s.e.m. **p<0.01 vs sham group. The ultrastructure of the cell was examined by transmission electron microscopy (TEM) after exposure to sham-radiation (**C**), 0.12 THz radiation with power of 10 mW for 30 min (**D**), or 0.157 THz radiation with power of 50 mW for 30 min (**E**). Organelles are indicated by arrows, green: nucleus; red: mitochondria; blue: ER; yellow: lysosome. Scale bars are 500 nm in **C,** and 2 μm in **D** and **E**.

The alteration of cellular activity might due to the changes of cell metabolism or cellular apoptosis. Therefore, we further analyzed the apoptosis by Annexin V and propidium iodide (PI) straining followed by flow cytometry. The results indicated that both 0.12 THz wave with power of 10 mW or 0.157 THz wave with power of 50 mW induced significant apoptosis (Fig 2A and 2B).

Then, the ultrastructure was observed by transmission electron microscopy (TEM). Sham-radiated cells showed nuclei with homogenous chromatin, intact organelles and very few damaged mitochondrial cristae (Fig 2C). Cells treated with power of 10 mW (0.12 THz) showed nuclei with homogenous chromatin. However, swollen mitochondria, damaged cristae, swollen endoplasmic reticulum and increased lysosome could be detected (Fig 2D). Cells treated with power of 50 mW (0.157 THz) showed similar changes but with fewer lysosomes (Fig 2E). These results suggested that both 10mW (0.12 THz) and 50 mW (0.157 THz) terahertz could cause induce structural injuries and result in cellular apoptosis.

### Terahertz radiation decreased the release of amino acid neurotransmitters in primary hippocampal neurons

**Fig 3:**
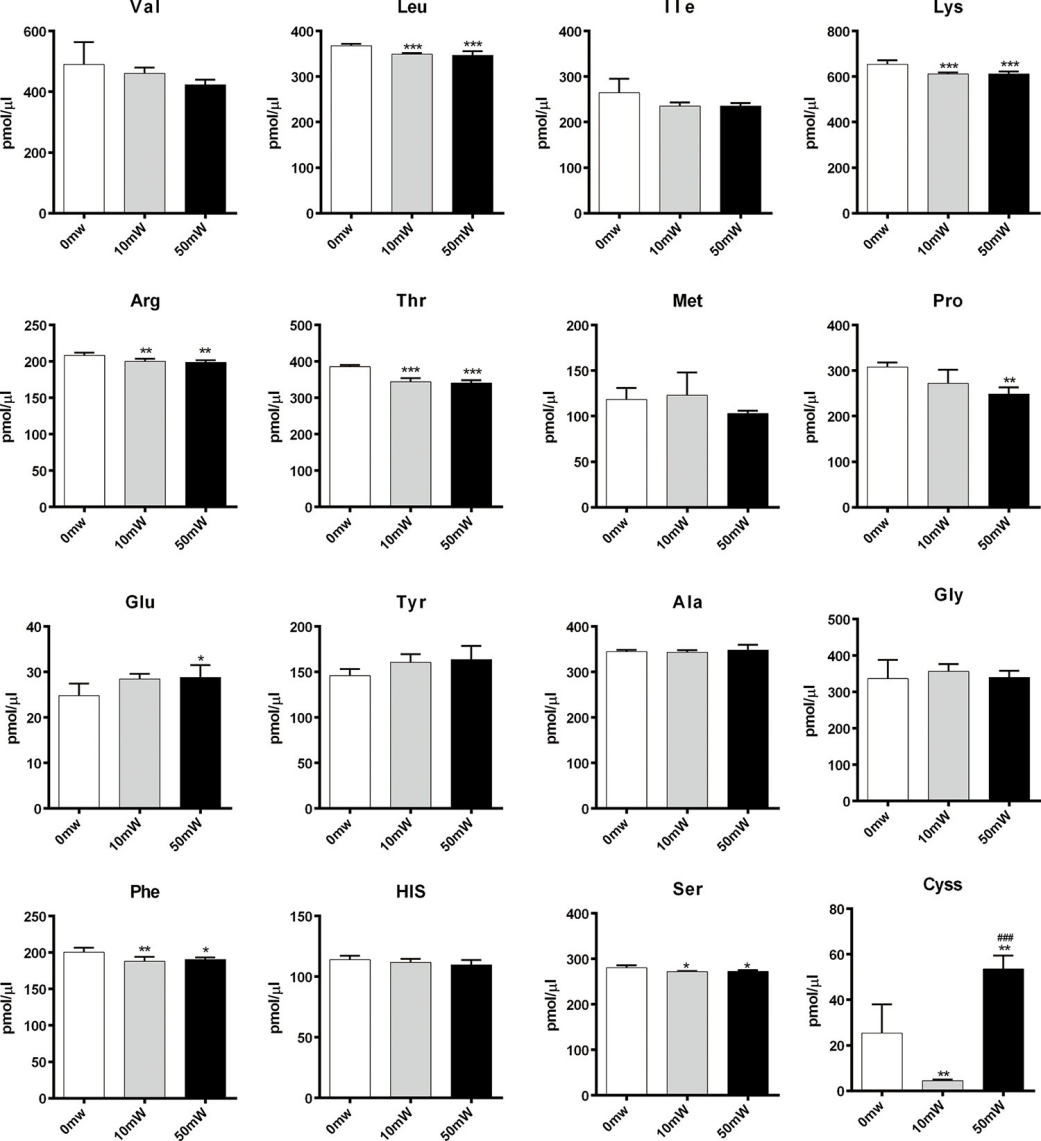
Effect of terahertz radiation on the release of amino acid neurotransmitters. Primary rat hippocampal neurons were radiated by terahertz wave at average power of 10 mW (0.12 THz) and 50 mW (0.157 THz) for 30 min. The culture media were collected and the concentrations of 16 amino acids were analyzed by HPLC. Sham-radiated cells were used as the controls. Data were shown as mean±s.e.m. *p<0.01, **p<0.01 and ***p<0.01 vs sham group; ^###^p<0.01 vs 10mW group.

Amino acid neurotransmitters are critical for transmitting nerve messages across the synapses. Some are inhibitory amino acids (IAAs) while others are excitatory amino acids (EAAs). The ratio of these amino acids could affect brain function [13]. In this study, we measured the concentrations of 16 amino acids in the sham-radiated and terahertz radiated primary hippocampal neurons by HPLC (Fig 3). In the culture medium, the concentration of Val, Ile, Met, Tyr, Ala, Gly and His was not affected by terahertz radiation, while the level of Leu, Lys, Arg, Thr, Phe and Ser was slightly down-regulated after exposure. Moreover, the Pro in 50 mW (0.157 THz) radiated cells was lower than those in other two groups, and statistical difference were detected in comparing the concentration of Pro. Furthermore, terahertz radiation caused an increase in the concentration of Glu and Cyss.

### Terahertz radiation had no obvious effects on expression of PSD95 protein

**Fig 4:**
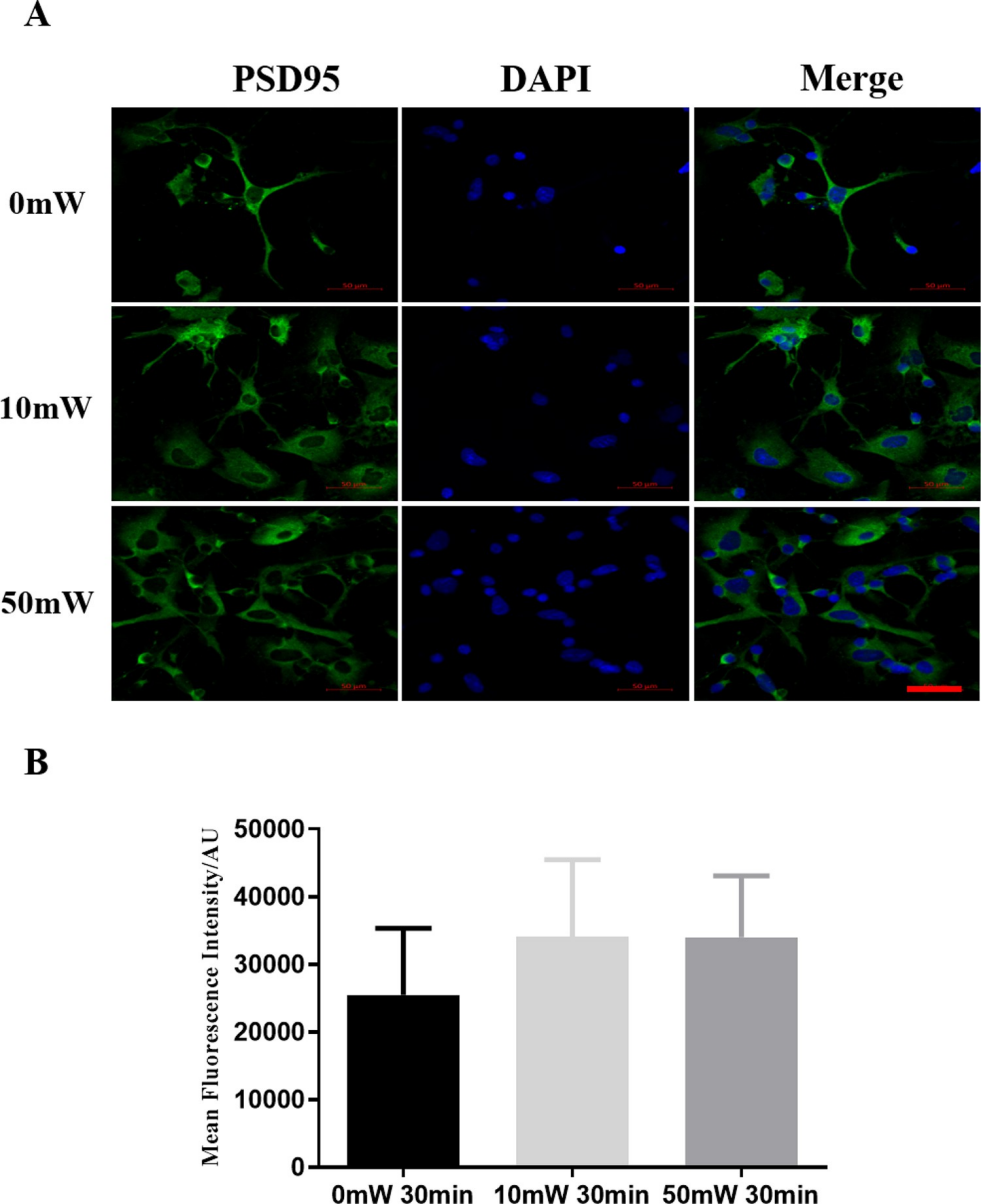
Expression of PSD-95 protein in neurons after exposure to terahertz radiation. Primary rat hippocampal neurons were exposed to terahertz radiation at an average power of 0, 10 mW (0.12 THz) or 50 mW (0.157 THz) for 30 min. The expression and localization of PSD-95 (green) was examined by immunofluorescence staining. Scale bar = 50 μm. Three independent experiments were conducted. The representative images were shown in **A**, and the statistical analysis were shown in B.

To determine whether terahertz radiation would affect neuron function, we examined the expression of postsynaptic density protein (PSD)-95, an important scaffold protein promoting synapse maturation, affecting synaptic strength and plasticity [14]. We observed the changes in the expression and localization of PSD-95 by immunofluorescence staining. The expression pattern of PSD-95 in terahertz radiated 10mW (0.12 THz) or 50 mW (0.157 THz)) cells did not differ significantly from that in the sham-radiated cells (Fig 4).

## Discussion

Terahertz has been widely used in braining imaging, however its potential effects on neuronal cells are still largely unexplored. In models of shellfish neurons and mollusk neurons, it has been reported that terahertz laser radiation caused damage to membrane integrity and membrane potential respectively [5, 15]. Some studies have found that specific terahertz wave frequencies can enhance the permeability of voltage-gated calcium channels [3], leading to an increase in intracellular Ca^2+^ concentration in neurons [7]. They can also lead to an increase in Glu and a decrease in Ala in primary cortical neurons [8], promoting the growth of ganglion processes [9]. But the effects of terahertz waves at different frequency and intensity on neurons have not been fully clarified. In this study, we investigated the effects of terahertz radiation on morphology, cellular activity, apoptosis, and the release of neurotransmitter in primary neuronal cells in vitro. We found that the effects are closely associated with the terahertz parameters, including power intensity, duration and wavelength. Generally, both 10mW (0.12 THz) and 50 mW (0.157 THz) terahertz radiation induced cellular apoptosis obviously in neurons companying with damaged mitochondria and increased lysosomes. However, low power intensity radiation (10mW, 0.12 THz) decreased cellular activity, while high power intensity radiation (50 mW, 0.157 THz) increased cellular activity. We speculated that the increased activity in primary neurons at immediately after radiation with high power intensity might be attributed to the responses to acute stress, throuth up-regulating calcium ions and enhancing enzyme activity [16]. Moreover, the contradictory results between low and high power intensity might associate with the differential gene expression. And, it has been demonstrated that terahertz radiation different terahertz intensities caused opposite gene expression patterns in mouse mesenchymal stem cells [17]. We are interested to explore the underlying mechanisms, which could be conducted by DNA or protein microarray, RNA-seq, ChIP-seq, and so on.

In this study, we showed that terahertz radiation affected the release of amino acid neurotransmitter by hippocampal neurons *in vitro*. Amino acid neurotransmitters are able to transmit messages across the synapse, and can be grouped into excitatory amino acids (EAAs) which could stimulate neurons activities, such as Asp and Glu, and inhibitory amino acids (IAAs)which could inhibited firing, such as Gly and GABA [18]. The balance between EAAs and IAAs is important for maintaining brain functions. Our data suggested that terahertz radiation could slightly increase Glu, an pivotal EAA of cognition, motor coordination and emotions [19]. And, several amino acids including Leu, Lys, Arg, Thr, Pro, Phe and Ser were down-regulated after terahertz irradiation. These amino acids are involved in cognitive behavior such as learning and memory. For example, nitric oxide (NO) which is synthesized from L-Arg has been implicated in the learning process and in memory formation [20]. Moreover, it has been reported that branched-chain amino acids (BCAAs), such as Leu could slightly improve brain functions both in animal experiments and clinical application [21]. In addition, BCAAs are precursors of Glu and GABA, which are critical to maintain balance of brain activities. L-proline can activate excitatory glutamate and inhibitory glycine receptors in cultured rat neurons [22]. It has been reported that patients with hyperprolinemia showed neurological disorders, including spatial memory deficit [23]. D-serine is a co-agonist of the N-methyl D-aspartate (NMDA) receptor to regulate NMDA receptor transmission, synaptic plasticity and neurotoxicity [24]. Threonine is a precursor of inhibitory neurotransmitter glycine. L-threonine increased glycine concentration in the rat central nervous system, as well as produced antispastic effect in human [25, 26]. Overall, exposure to terahertz radiation could alter the release of amino acid neurotransmitters, which might cause negative effects, such as increased anxiety on mouse behaviors [4].

In addition to neurotransmitters, the balance between neuronal excitation and inhibition is also could be regulated by molecules that control the assembly of neurotransmitter receptors, signal transduction and synapse maturation. PSD-95, containing 3 PDZ domains, an SH3 domain and a guanylate kinase region, belongs to the membrane-associated guanylate kinase family [27, 28]. PSD-95 has an important role in regulating protein trafficking and ion channel clustering, particularly on glutamate receptor clustering and function. It binds to the NR2 subunit of NMDA-type glutamate receptors and modulates NMDA receptor clustering, activity and signaling [29]. PSD-95 could not only enhance AMPA recruitment and excitatory synaptic response [30], but also interact with other molecules to regulate synapse morphology, maturation and plasticity [31]. In this study, we found that terahertz radiation does not affect the expression or localization of PSD-95, indicating that terahertz radiation modulated synaptic functions through a PSD-95 independent mechanism.

## Conclusions

The effect of terahertz radiation on rat hippocampal neurons in vitro are closely related with the parameters of radiation. Generally, terahertz induced apoptosis and altered cellular activity of primary hippocampal neurons, as well as regulated release of amino acid neurotransmitter, which in turn affected the homeostasis of neuronal excitability.

## Supporting information

S1 File (DOCX)
