# Supplementary figures and images for: Biological responses to terahertz radiation with different power density in primary hippocampal neurons
Source: PLoS One. 2023 Jan 20;18(1):e0267064. doi: 10.1371/journal.pone.0267064 (PMC9858065; doi:10.1371/journal.pone.0267064)

**Figure 1**


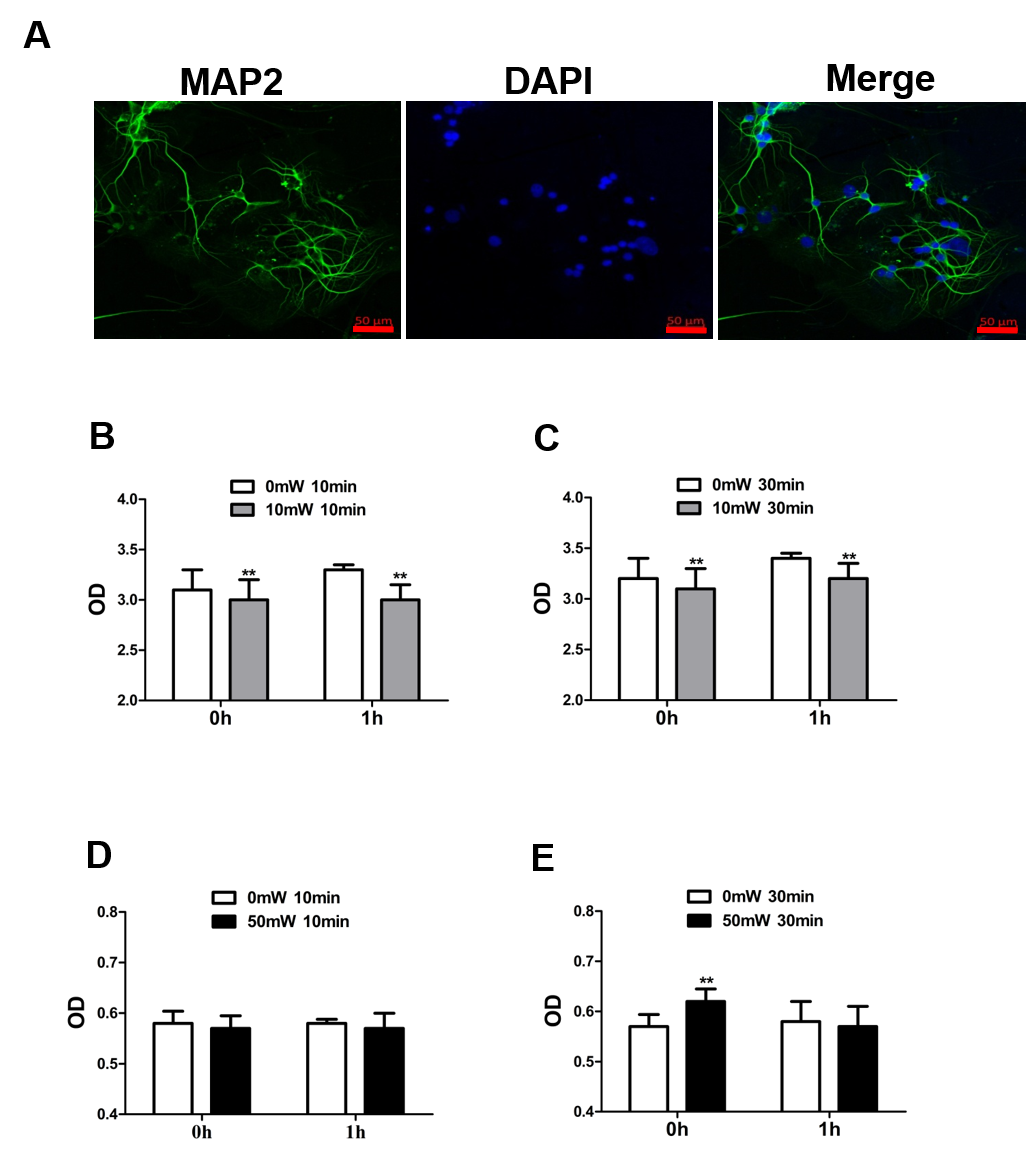


**Figure 2**


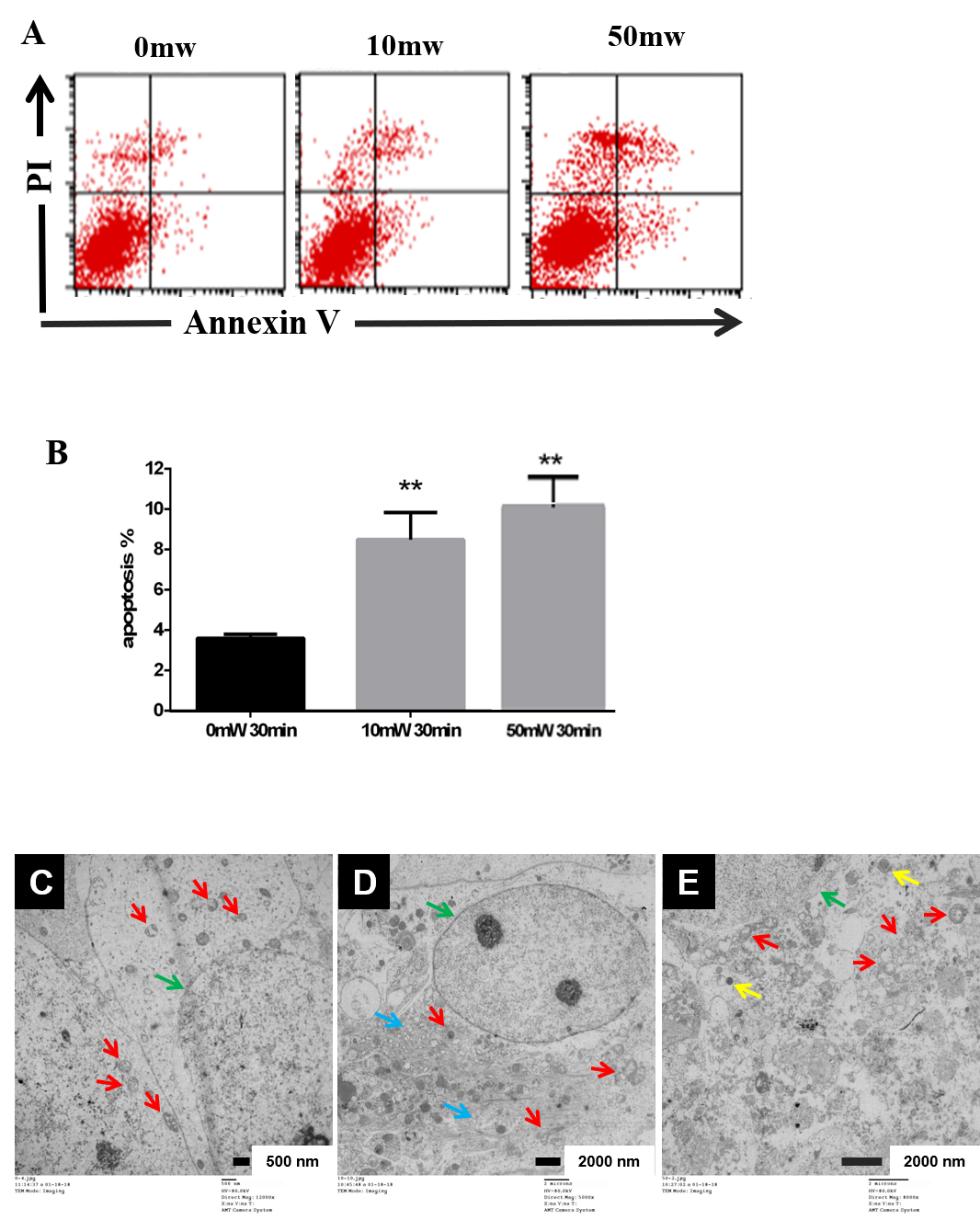


**Figure 3**


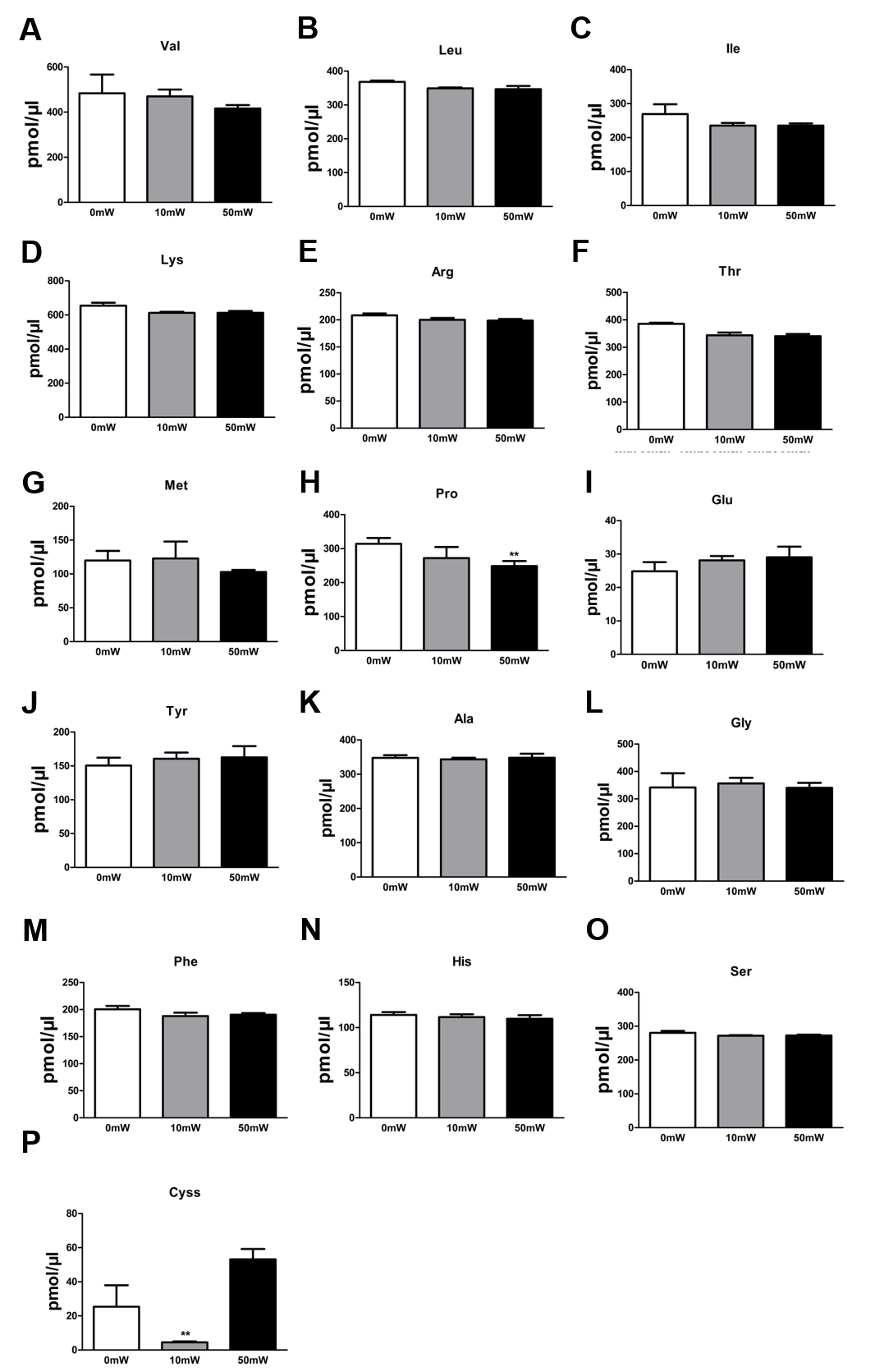


**Figure 4**


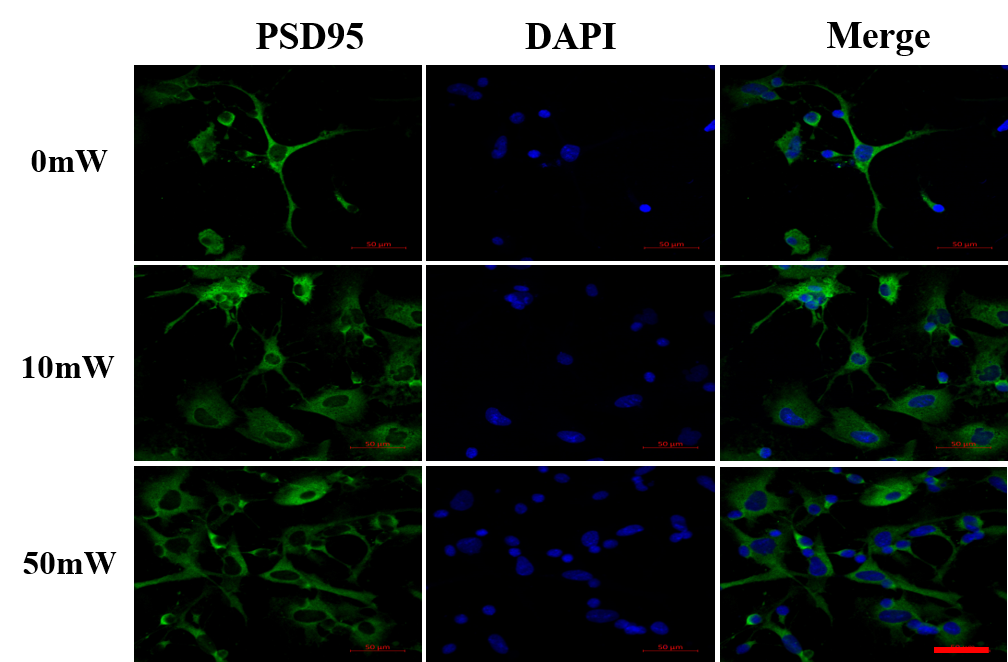

Supplement: S1 File — (DOCX) [file pone.0267064.s001.docx]
